# Intermittent fasting in the treatment of type 2 diabetes

**DOI:** 10.3389/fnut.2025.1629154

**Published:** 2025-08-18

**Authors:** Damian Dyńka, Łukasz Rodzeń, Mateusz Rodzeń, Dorota Łojko, Adam Deptuła, Żaneta Grzywacz, Sebastian Kraszewski, Karolina Bartoń, Peter Martin, Anna Małgorzata Deptuła, Ken Berry, David Unwin

**Affiliations:** ^1^Institute of Health Sciences, Faculty of Medical and Health Sciences, University of Siedlce, Siedlce, Poland; ^2^Rodzen Brothers Foundation, Wieleń, Poland; ^3^Department of Psychiatry, Poznan University of Medical Science, Poznan, Poland; ^4^Faculty of Production Engineering and Logistics, Opole University of Technology, Opole, Poland; ^5^Department of Biomedical Engineering, Faculty of Fundamental Problems of Technology, Wroclaw University of Science and Technology, Wroclaw, Poland; ^6^Karolina Bartoń Food Coach, Warsaw, Poland; ^7^Funmed Clinics, Vastra Hamngatan, Gothenburg, Sweden; ^8^Independent Researcher, Holladay, TN, United States; ^9^Faculty of Health Social Care and Medicine, Edge Hill University, Ormskirk, United Kingdom

**Keywords:** type 2 diabetes mellitus (T2DM), intermittent fasting (IF), insulin resistance, glucose, insulin, body weight, novel technologies, artificial intelligence

## Abstract

The increasing prevalence of type 2 diabetes mellitus (T2DM) has inspired researchers to investigate therapies and tools supporting the treatment of this disease. One such tool is intermittent fasting (IF). Given the nature and mechanism of action of IF, it would be logical for it to have a beneficial effect on T2DM patients. This study analyzes the role of IF in the treatment of type 2 diabetes, used alongside standard recommendations, based on the current literature available to the authors. The authors discuss the benefits of IF in T2DM treatment, such as improved glycaemic control, improved insulin sensitivity, facilitated adherence to recommendations, weight reduction, and lower risk of complications. This study covers the results of meta-analyses, systematic reviews, and randomized controlled trials (RCTs) and shows how novel technologies, including continuous glucose monitors and mobile applications, can support the implementation of IF. The importance of safety monitoring is also highlighted, particularly in insulin-treated patients due to the potential risk of hypoglycaemia.

## Graphical abstract

Created in BioRender. Rodzeń (2025) https://BioRender.com/er69usg. 
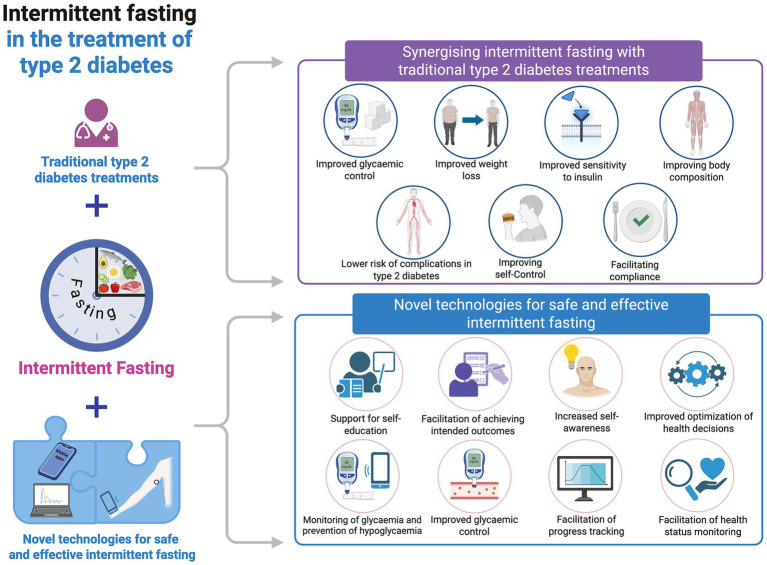

## 1 Introduction

Type 2 diabetes mellitus (T2DM) and intermittent fasting (IF) are two distinct topics, commonly investigated by researchers. Although both have been known for centuries, only recently have we seen a significant increase in papers addressing the two topics simultaneously (1, 2). It is paradoxical that despite the progress in understanding type 2 diabetes, the prospects (and costs) associated with the disease (described in Chapter 3) for the coming years are alarming (3–6). Therefore, greater effort in investigating various tools for the treatment of T2DM is important. If used properly, intermittent fasting may be one such tool. We hope to show that with more focused research and accelerated development of novel technologies, IF can be a very easy and safe practice. This is of particular importance in insulin-treated type 2 diabetes, where inter alia 24-h blood glucose monitoring is essential (7, 8). Given the characteristics of the disease on the one hand and the results of many scientific studies looking at the effects of intermittent fasting on the other, the combination of the two seems to be synergistic. The issue has even been addressed in the latest edition of “Standards of Care in Diabetes – 2024” (9), which explicitly suggests that intermittent fasting can be a helpful tool, compatible with any diet and safe to use in both types of diabetes. The authors of these standards also point to the importance of blood glucose monitoring of diabetic patients practicing intermittent fasting. The 2024 Standards also address the possible role of “chrononutrition” (how the timing of food ingestion affects metabolic health), which has much in common with intermittent fasting as it relates to the timing of meals (10–13). This makes time-restricted eating an accepted and even recommended tool in the treatment of T2DM. As intermittent fasting is completely free to use, it may also be the cheapest of all available therapeutic tools. The objective of this study is to evaluate IF in treating type 2 diabetes, based on an analysis of the scientific research known to the authors. In addition, the study shows how novel technologies can be helpful in practicing intermittent fasting in T2DM. Indirectly, it also aims to highlight the indisputable role of lifestyle in the prevention and treatment of type 2 diabetes.

## 2 Fasting and intermittent fasting

### 2.1 Fasting

Fasting can be defined as “time-limited abstention from solid foods and caloric fluids, in order to suspend (or minimize) energy supply to the body.” The duration of this period is not strictly defined; however, Longo and Mattson note that it usually ranges from 12 h to 3 weeks (14, 15).

The longest known period of fasting is believed to be endured by Angus Barbieri, who, under medical supervision, consumed no foods or caloric fluids (and therefore no calories) for 382 days. According to the authors of his case study, “Prolonged fasting in this patient had no ill-effects.” Importantly, however, at the start of the experiment, Mr. Barbieri’s body weight was 207 kg, 125 kg of which he ultimately lost. Thus, a vast amount of energy stored in the form of body fat was available to him (16, 17). This means that for the vast majority, such long fasting may not be safe.

The body’s adaptation to the fasting state is probably a form of ancestral evolutionary adaptation that helped the human species to survive (18). Among other things, it involves the breakdown of stored glycogen and proteins to produce glucose, alongside the use of stored fat to produce ketone bodies from fatty acids released into the bloodstream (19–21). Fasting is also completely natural in animals, as it directly reflects the environmental conditions they live in. In addition, fasting has been observed in sick animals, which is caused by loss of appetite, among other things (17, 22). Loss of appetite is also commonly observed in humans suffering from a wide variety of conditions (23).

Since antiquity, fasting for medicinal purposes has been practiced in many cultures. Hippocrates, the founding father of modern medicine, wrote “To eat when you are sick, is to feed your illness.” In his turn, about 500 years ago, Philip Paracelsus believed that “Fasting is the greatest remedy-the physician within” (24–26). Other philosophers who praised the benefits of fasting include Socrates, Aristotle, Plato, and Galen (27). In one way or another, fasting is present in all major religions. This includes the periods of Lent and Advent in Christianity, Yom Kippur in Judaism, Ramadan in Islam, Ekadashi in Hinduism, or the month of Alá in Bahaism. Some Buddhists (in addition to abstaining from certain foods in general) abstain from food in the afternoon, which is a form of intermittent fasting (17). Some forms of fasting do not entail complete abstinence from food, but rather involve the restriction of specific dietary components, such as grains (e.g., wheat and rice) and legumes, as is the case during the religious fast of Navratri observed by Hindus. Nonetheless, even these selective dietary restrictions have been associated with beneficial metabolic effects, including significant reductions in HbA1c levels and blood glucose concentrations (28).

The first recorded references to fasting date as far back as the 5th century BC, and until 1921, the practice was used to treat epilepsy (29, 30). Fasting works by inducing ketosis in the body, a state in which ketone bodies are produced from fats (ingested and/or broken down). Subsequently, ketone bodies become the body’s main source of energy, just like in people on a ketogenic diet (31). Fasting is described even in the scriptures (Mark 9:29), where Jesus healed a boy of epilepsy and suggested that the condition could be cured with fasting and prayer alone “That kind of spirit comes out only if you use prayer and fasting” (32–34). Fasting for this purpose was discontinued in 1921, when it was discovered that ketosis could also be induced by the ketogenic diet, which, unlike fasting, could be used in the long term without the negative effects of starvation (35, 36).

### 2.2 Intermittent fasting

Fasting is a relatively broad concept, and intermittent fasting (IF) is one of its most common forms. Intermittent fasting is the abstinence from food and caloric fluids over a specific period of time, followed by a period of food consumption (24, 37). According to the authors of the 2022 publication, IF can be divided into two main categories: fasting during the week and fasting during the day. Fasting during the week is further divided by those authors into Alternate-Day Fasting (ADF), which means alternating ad libitum food intake for 24 h followed by 24 h of fasting, and Twice-Weekly Fasting (TWF), with 2 days of fasting per week and ad libitum consumption in the remaining 5 days. Conversely, fasting during the day involves consumption of meals in a time window of 4–10 h (usually 8) and fasting for the rest of the day. If the feeding window is in the morning, the authors refer to this as Early Time-Restricted Eating (eTRE), and if it occurs later in the day, the term to describe the practice is Delayed Time-Restricted Eating (dTRE) (38). Importantly, however, intermittent fasting is a very fluid concept. It seems that the only common denominator is fasting for a certain period of time, alternating with a period of food intake.

Currently, an extensive body of scientific literature exists to illustrate the beneficial effect of intermittent fasting on metabolic health (24, 39, 40). A number of meta-analyses and systematic reviews confirm that IF has beneficial effects on, among other things, weight loss and chronic disease risk parameters (41), including cardiovascular risk factors in patients with the metabolic syndrome (42), reduction in depression rates (43), richness of the gut microbiota (44), potential benefits in the treatment of multiple sclerosis (45) and non-alcoholic fatty liver disease (NAFLD) (46), improved body composition (47), or specific benefits for patients with type 2 diabetes (48).

It is important to clearly distinguish between intermittent fasting (IF) and starvation. Intermittent fasting does not inherently imply a caloric deficit, unlike starvation, which by definition involves prolonged energy and nutrient deprivation. Starvation has been shown to have long-term physiological consequences, particularly a persistent reduction in metabolic rate, which may remain suppressed long after normal eating resumes and, in some cases, may never fully recover (49). In contrast, as demonstrated in later sections of this article, IF has been associated with a range of health-promoting effects in various contexts.

## 3 Insulin resistance and type 2 diabetes

### 3.1 Insulin resistance

Over the past decades, insulin resistance has emerged as a major risk factor in many chronic diseases, such as type 2 diabetes (50, 51), cardiovascular diseases (52), non-alcoholic fatty liver disease (NAFLD) (53), and even cancer (54). Insulin resistance significantly affects public health. In the US, it is estimated to occur in as many as 40% of adults aged 18 to 44 years, based on HOMA-IR values (55). Since fasting insulin (among other things) is not tested routinely despite the prevalence of insulin resistance, a significant proportion of people may go undiagnosed, and the condition may progress gradually for years. Meanwhile, insulin resistance is known to lead primarily to type 2 diabetes (56–58). It is therefore possible that even people with advanced diabetes are still unaware of the disease. This is confirmed by official statistics, which estimate that in the United States alone, 8.7 million people, or 22.8% of all individuals with diabetes, remain undiagnosed (59). With this in mind, it is reasonable to believe that early diagnosis of insulin resistance could often help prevent diabetes and other conditions associated with it.

Insulin resistance itself is otherwise known as impaired cellular sensitivity to insulin, the hormone responsible for allowing glucose from the blood into cells. Liver, skeletal muscle, and adipose tissue cells are particularly affected, but all cells containing insulin receptors can become resistant to the hormone (50, 60). Under normal conditions, the body breaks down ingested carbohydrates into glucose, which is subsequently released into the bloodstream. The beta cells of the pancreas then secrete insulin, which lowers blood glucose levels by moving it into the cells. When the insulin receptors are activated, they undergo autophosphorylation of tyrosine residues and trigger intracellular signaling pathways, including the phosphatidylinositol-3-kinase (PI3K) and protein kinase B (PKB/Akt) pathways. The PI3K/Akt pathway leads to the translocation of GLUT4 glucose transporters from internal stores to the cell membrane. GLUT4 in the cell membrane enables glucose transport from the blood into the cells (61). PTEN, a lipid phosphatase, functions as a key negative modulator of the PI3K/Akt signaling cascade, an essential pathway in insulin-mediated metabolic regulation. By dephosphorylating PIP3 back to PIP2, PTEN counteracts Akt activation, thereby attenuating insulin signaling. Increased expression or activity of PTEN has been associated with impaired glucose uptake and disrupted metabolic homeostasis, ultimately contributing to the development of insulin resistance. The inclusion of this molecular mechanism could enrich the section on insulin resistance (62). In insulin resistance, impaired cellular sensitivity to insulin forces the pancreas to produce ever more insulin (hyperinsulinaemia) to regulate the flow of glucose from the blood into the cells and maintain homeostasis. The normal serum glucose level is sustained only until the pancreatic production of insulin is capable of compensating for the cells’ resistance to the hormone. With increased insulin resistance, the baseline blood glucose level grows due to the deficiency of GLUT4 transporters (50, 63). The underlying causes of impaired insulin action are complex. They originate from a number of factors, such as overeating, obesity, chronic inflammation, energy imbalance, excess free fatty acids in the bloodstream, abnormal adipokine levels that affect, among other things, the sensation of hunger, or dysfunction of the mitochondria themselves (64). When endogenous insulin production is insufficient to compensate for increasing insulin resistance, serum glucose levels go up leading to a pre-diabetic state (fasting glucose levels of 100–125 mg/dL and A1C 5.7–6.4%) and consequently to diabetes (fasting glucose levels ≥126 mg/dL and A1C ≥ 6.5%) (50, 65).

### 3.2 Type 2 diabetes

Type 2 diabetes is a chronic metabolic disease characterized by inefficient use of insulin produced by the body (as opposed to type 1 diabetes, whereby insulin production is impaired due to damage to the beta cells of the pancreas, among other things). Type 1 diabetes is far less common and represents only 5–10% of all diagnosed cases of diabetes. Unlike T2DM, it is much more frequent in children than in adults. Type 2 diabetes can be seen as a further consequence of insulin resistance and pre-diabetic status (and even a condition indirectly associated with obesity). Diabetes is a major cause of all-cause mortality from heart attacks, strokes, blindness, kidney failure, and even lower limb amputations, among others (4, 66, 67). Recently, there has been a transition from vascular causes to cancers as the leading contributor to death rates in individuals with T2D (68).

The prognosis of the disease is alarming. A 2023 paper found that in 2021, 529 million people worldwide had diabetes and warned that over 1.31 billion patients could be affected by 2050 (69). The projection is much more troubling than one made only a few years earlier by Saeedi et al. (70), who expected that the increase would be from 460 million to 700 million in 2045.

In the US, the cost of diagnosed diabetes has been estimated at $412.9 billion ($306.6 billion in direct costs and $106.3 billion in indirect costs), and this is for 2022 alone. The disease is believed to have become a serious challenge to healthcare systems, which are severely burdened by the direct and indirect costs of its treatment (3). European statistics, on the other hand, indicate that $189 billion was spent on diabetes in 2021 (71). Another team of authors estimated that in China, the total costs of diabetes would increase from $250.2 billion in 2020 to $460.4 billion in 2030, thus significantly increasing the economic burden (6). In Poland, the costs of diabetes-related medical services, devices, and drugs alone grew from PLN 1.9 billion in 2018 to PLN 2.5 billion in 2022. Meanwhile, it is estimated that by 2030, 1 in 10 people in Poland will have diabetes (compared to 3.11 million Poles in 2022) (72).

In summary, current treatments are not sufficient; new methods and tools should be developed and, once validated, included in standard diabetes treatment regimens.

## 4 Synergizing intermittent fasting with traditional type 2 diabetes treatments

Although it would seem logical that drug-free remission should be the long-term goal of type 2 diabetes treatment (which is possible) (73, 74), the officially defined primary goal of treatment is to control hyperglycaemia (i.e., to keep glucose levels within a low diabetic range) and to treat the disease’s comorbidities (such as hypertension) (75, 76). It appears that intermittent fasting may help to achieve not only these goals but also a number of others that are extremely important in the treatment of type 2 diabetes. The combination of traditional T2DM treatment with intermittent fasting may offer a kind of synergy, resulting in better therapeutic outcomes. The range of the potentially synergistic effect of the two approaches is described in the following subsections.

### 4.1 Improved glycaemic control

The stabilization and control of serum glucose is by far the most important aspect in the treatment of type 2 diabetes (75, 76). To this end, the most recent “Standards of Care in Diabetes-2024” recommends, inter alia, replacing sugar-sweetened beverages (including fruit juices) with, for example, water, choosing non-starchy vegetables, nuts, unprocessed carbohydrates, and whole-grain and fibre-rich foods. Importantly, however, the importance of reducing total carbohydrate intake is being increasingly appreciated. Apparently, most evidence links glycaemic improvement with the reduction of total carbohydrates (77). The emphasis on patient education regarding the effects of carbohydrates, proteins, and fats on glycaemia is another sound argument presented in the standards (9, 78). It seems reasonable that if carbohydrates (even those from whole-grain products) are the main macronutrient stimulating blood glucose release, then limiting their supply will significantly reduce post-meal glucose peaks. Patient education in this regard is therefore extremely important and probably still underestimated.

Intermittent fasting can be effective in controlling glycaemic levels, as they tend to stabilize in periods of food abstinence, free from glucose spikes that accompany every meal (especially those rich in carbohydrates). A number of academic publications confirm the benefits of fasting in this regard. One systematic review with meta-analysis concluded that IF is effective in patients with disorders of glucose and lipid metabolism. Based on a number of studies, it was demonstrated that IF improved several parameters, such as mean glucose, insulin, glycated haemoglobin (HbA1c), and HOMA-IR. Remarkably, as noted by the authors themselves, IF did not involve strict calorie limitations in any of the studies (79). Improved glycaemic levels are also confirmed by a randomized controlled trial (RCT) by Jamshed et al. (80). They found that fasting for most of the day and eating only between 10:00 and 14:00 (early TRF, eTRF) was more efficient in reducing glucose levels (by an average of 4 ± 1 mg/dL) and blood sugar spikes (by an average of 12 ± 3 mg/dL) per day compared to eating between 8:00 and 20:00 (80). Another RCT compared the effects of intermittent energy restriction (IER) with continuous energy restriction (CER). One of the analyzed parameters was HbA1c in patients with type 2 diabetes. The study found that both groups improved their glycaemic control and were able to reduce medication dosage in just 12 weeks (81). This finding is well in line with the results of a meta-analysis of studies involving type 2 diabetes patients that assessed the safety of intermittent fasting compared to continuous energy-restricted diets (CERD). Its authors demonstrated that intermittent fasting is as effective in glycaemic control and managing fasting insulin values as continuous calorie restriction. In addition, it is more efficient in terms of weight loss. The authors of the meta-analysis explicitly concluded that IF is safe and can be used in patients with type 2 diabetes or metabolic syndrome (82). This means that if used in combination with standard recommendations (as they are not contradictory to IF), fasting may work synergistically, improving glycaemic control in T2DM patients. It is also worth noting that glycemic variability may induce a greater degree of apoptosis and a more pronounced decline in insulin secretory function of pancreatic beta cells compared to sustained hyperglycemia (83). Intermittent fasting, through its potential to stabilize blood glucose levels, may help mitigate this effect. However, it should be emphasized that poorly implemented intermittent fasting protocols, or the initial phase of adaptation to IF, may transiently increase glycemic fluctuations before stabilization occurs. The overall impact of intermittent fasting on aspects such as glycaemic levels in the diabetic population is discussed in Chapter 5.

### 4.2 Improved sensitivity to insulin

Since insulin resistance is the underlying cause of type 2 diabetes, improving tissue insulin sensitivity seems to be essential in obtaining improvement or even remission. Officially, improving insulin sensitivity is attempted by means of medication, mainly metformin, recommended even for prevention, given the increasing insulin resistance in the pathogenesis of diabetes (73, 84).

Intermittent fasting may offer specific benefits in terms of sensitizing cells to insulin action. It is known that fasting eliminates postprandial glucose spikes and, consequently, insulin secretion from pancreatic *β*-cells. The beneficial effect of IF on insulin sensitivity has been demonstrated in many studies. An early human study took place in 2005, with a group of eight healthy individuals practicing intermittent fasting for 20 h every other day for 15 days. The objective was to test the effect of intermittent fasting on insulin sensitivity (85). Before and after the intervention, euglycemic hyperinsulinaemic clamps were performed, and the glucose infusion rate (GIR) and glucose levels were compared. The study found that the insulin-mediated glucose uptake increased from 6.3 ± 0.6 to 7.3 ± 0.3 mg/min/kg after IF, even though body weight was not significantly different at the two time points. The authors themselves noted that this was the first human clinical study to show that IF accelerates insulin-mediated glucose uptake. A 2022 systematic review of clinical trials has shown that in patients with metabolic disorders, IF can help reduce insulin resistance, improve glucose and lipid metabolism, and reduce body weight, thus being an effective therapeutic option in these patients (86). Another systematic review of clinical trials showed that the inclusion of IF in patients with metabolic syndrome was followed by reduced insulin resistance, with significant mean level reduction for insulin (by 13.25 uUI), glucose (by 0.15 mmoL/L), and HOMA-IR (by 0.31). Body weight loss was also observed. Based on the analyzed studies, the authors concluded that IF significantly reduces insulin resistance. They openly suggest that intermittent fasting can be considered as an adjunctive treatment to prevent the onset and progression of chronic diseases (79). A 2021 extensive review of the literature looked at studies analyzing the correlation between IF on the one hand and insulin resistance and type 2 diabetes on the other. The review found that most of the available studies demonstrated IF-mediated reduction in insulin resistance. Interestingly, in some studies, IF could even eliminate the need for insulin supplementation. The authors conclude that IF is an effective, non-medical option for treating type 2 diabetes. They also point out that if the goal of intermittent fasting is to reduce or even eliminate diabetes medication (including insulin), then it should only be practiced under the close supervision of a physician (87). The advice for a physician to monitor IF in type 2 diabetes and the likely reduction or withdrawal of medication demonstrates that intermittent fasting can be an extremely powerful therapeutic tool in this disease. This in itself is yet another argument showing that the combination of traditional treatment and recommendations can synergize with properly practiced intermittent fasting, significantly improving the health and quality of life for people living with diabetes. Potentially reducing dependency on medication also reduces the risk of side effects. The effect of IF on insulin levels in patients with type 2 diabetes is also discussed in Chapter 5. Insulin, along with HOMA-IR and leptin levels, undergo changes during intermittent fasting (IF) (88, 89), suggesting that these biomarkers may play a role in guiding or tailoring IF interventions to individual patient profiles. While this remains an emerging area of interest, these biomarkers have the potential to enhance the personalization of IF therapy, thereby improving both efficacy and safety in patients with various metabolic disorders.

### 4.3 Facilitating compliance

Nutritional recommendations are ineffective unless they are correctly implemented and adhered to. Hence, compliance may be crucial and instrumental in whether the patient achieves the desired goal or gives up early. Meanwhile, adherence to dietary recommendations among people with type 2 diabetes is very low (90). A study by Baral et al. (91) found that only 15.7% of patients with T2DM adhered to the recommendations. The authors indicated that affordability (along with self-monitoring and physical activity) is one of the key factors affecting dietary compliance in these patients (91).

Intermittent fasting may fit well with the recommendations for diabetes and make them easier to adhere to. With a view to its simplicity, the most recent standards explicitly suggest IF as a practical, useful tool for people with type 2 diabetes (9). This is particularly relevant in the context of reducing the number of meals per day. Much evidence suggests that a high number of meals per day is not the best recommendation, and lowering that number may even reduce the risk of T2DM (92). The authors of a 2024 systematic review found that two to three meals per day combined with intermittent fasting (which means the daily eating window is shorter than 10 h) promotes, among other things, glycaemic control and weight loss in patients with type 2 diabetes (93). The authors of another very interesting study concluded that new approaches, going beyond those used so far, should be explored to help reduce the excessive burden caused to healthcare systems by lifestyle diseases. They therefore set out to test the effectiveness of TRE intermittent fasting on 63 volunteers, who were asked to reduce their daily eating window to 8 h for 3 months. TRE was found to be a viable option, acceptable even for people with a normal working life. In addition, it significantly improves health-related quality of life (HRQoL) and prevents lifestyle diseases (e.g., owing to weight loss) (94). Combining the practicality, efficacy, and simplicity of intermittent fasting with a standard therapeutic approach may therefore work synergistically to facilitate compliance and thus the effectiveness of type 2 diabetes treatment.

### 4.4 Fasting, self-control, and metabolic health—a historical and psychosocial insight

In addition to simplifying meal routines, intermittent fasting may enhance adherence by improving self-regulation. Ancient texts, including the Bible, reference fasting not only as an abstention from food, but as a structured act of restraint and inner regulation (e.g., Daniel 1:12–16). This aligns with current scientific observations that intermittent fasting supports improved adherence (95), enhances metabolic flexibility (96), and promotes neuroendocrine stability (97). Studies indicate that intentional dietary restriction activates brain regions linked to executive function and impulse control (98), while reducing circulating levels of cortisol and inflammatory markers (99). The psychosocial aspects of fasting—rooted in discipline, routine, and delayed gratification—may contribute to better long-term glycaemic control and reduced emotional eating, which is commonly observed in patients with type 2 diabetes (100). Moreover, regular engagement in structured fasting practices is associated with favorable changes in leptin and adiponectin levels, improved insulin sensitivity, and lower HOMA-IR scores, independent of caloric intake (101).

### 4.5 Weight loss

Losing excess body weight is one of the goals that should be pursued by overweight and obese people with type 2 diabetes. It offers a number of beneficial outcomes in terms of glycaemic control, blood pressure, or lipid levels (9).

Intermittent fasting may help in weight loss, if only by facilitating compliance (see section 4.4 for details) and shortening the daily eating window. In the aforementioned study by Kesztyüs et al. (94) participants lost weight despite not having to count calories. This is extremely important, as a shorter eating window itself promotes the consumption of smaller quantities of food per day. A 2024 meta-analysis has shown that in obese individuals over 40 years of age, IF reduced body weight and fat mass. These effects were not accompanied by lean tissue loss, which is highly beneficial (102). Studies show that IF is as effective for weight loss as continuous calorie restriction (103, 104). A systematic review of 27 studies found that IF resulted in weight loss of between 0.8 and 13% vs. baseline. Among patients with type 2 diabetes, it additionally improved glycaemic control (105). In 2022, the results of a meta-analysis comparing the effectiveness of IF and continuous calorie restriction (CCR) were published. The authors concluded that, in some respects, IF may even be superior to CCR in terms of weight loss (106). This indicates that intermittent fasting may be an effective weight loss strategy and, to some extent, may outperform standard continuous calorie restriction. In patients with type 2 diabetes, IF was also associated with greater weight loss than continuous energy-restricted diets (CERDs) (82). A 2024 systematic review further demonstrated that IF improved body composition without reducing physical performance. It may therefore be recommendable even for athletes wishing to maintain lean body mass while losing body fat (107). Additionally, intermittent fasting may exert beneficial effects on obesity by modulating the gut microbiota profile and the production of gut-derived metabolites, as well as by reducing inflammation and contributing to glycaemic regulation mechanisms that are increasingly highlighted in the scientific literature (108). Given the above, the synergy of IF with standard recommendations may improve weight loss, which is crucial in patients with type 2 diabetes who often struggle with overweight or obesity.

### 4.6 Muscle mass preservation and physical activity during fasting

A frequent concern regarding IF is the potential loss of lean body mass. However, current evidence from clinical trials suggests that short-term fasting, when properly implemented, does not result in significant muscle loss. On the contrary, fat loss tends to be predominant, while lean mass is preserved or even improved. A randomized controlled trial involving 70 days of alternate-day fasting (ADF) showed no measurable decline in fat-free mass despite substantial fat loss (109). In another RCT lasting 32 weeks, participants following an intermittent fasting protocol lost less lean tissue than those on daily caloric restriction, and they experienced a greater increase in lean mass percentage relative to total body weight (2.2% vs. 0.5%) (110). These outcomes are likely supported by hormonal changes that occur during fasting, including increased secretion of growth hormone, which promotes fat oxidation while preserving protein structures (111). Unlike continuous caloric restriction, intermittent fasting does not significantly suppress resting metabolic rate, further preserving energy balance and metabolic resilience (110). Incorporating resistance or strength training into fasting protocols enhances these protective effects on muscle tissue, supports glucose disposal, and improves insulin sensitivity—all key factors in managing type 2 diabetes mellitus.

Moreover, physical activity promotes weight loss and enhances insulin sensitivity, thereby contributing to the reduction of blood glucose levels (112). Intermittent fasting (IF) has also been associated with improved glycaemic control and body weight reduction. Therefore, combining IF with regular physical activity may exert a synergistic effect, potentially yielding greater metabolic benefits for individuals with type 2 diabetes. This notion is supported by studies demonstrating that the integration of IF with physical exercise leads to superior outcomes compared to IF alone, particularly in terms of greater reductions in fat mass, waist circumference, insulin levels, HOMA-IR (Homeostasis model assessment of insulin resistance), and LDL cholesterol (113). Additionally, a recent meta-analysis confirmed that IF, whether hypocaloric or eucaloric, can be effectively combined with physical training without compromising most measures of physical fitness, while significantly enhancing weight loss and obesity-related outcomes (114).

### 4.7 Lower risk of complications in type 2 diabetes

One of the main goals in type 2 diabetes management is to mitigate and prevent its complications, often related to the cardiovascular system. Increased risk of cardiovascular disease (CVD) is already present in the pre-diabetic state, and its level closely reflects the progress of the disease (75, 84, 115). The major cause of morbidity and mortality in people with diabetes is broadly defined as atherosclerotic cardiovascular disease (ASCVD) and heart failure. It is estimated that as much as $39.4 billion is spent annually on managing diabetes-related cardiovascular conditions (3, 116).

Intermittent fasting may be beneficial in reducing the cardiovascular complications of diabetes. In 2024, a comprehensive systematic review found evidence to suggest the benefits of fasting, including IF, in reducing the population risk of cardiovascular disease. The authors point to the effects of fasting (including IF) on improving lipid profile, metabolic syndrome markers and inflammation, lowering body weight, and reducing insulin resistance, among other things (37). One study even concluded that IF (in the form of Ramadan) improved endothelial and non-endothelial dependent vasodilation and lowered blood pressure (117). Another similar study showed that intermittent fasting can have a positive effect on vascular endothelial function and even modulate cardiovascular risk (118). Findings from a more recent 2023 study confirm that 30 days of IF lead to an improvement in endothelial function, associated with improved flow-mediated dilatation (FMD). In addition, IF lowered CRP (C-reactive protein) and cortisol levels (which may contribute to improved FMD) (119). The authors of another publication from the same year conclude that intermittent fasting may prevent atherosclerosis and point to its multi-layered benefits. They indicate that IF reduces resistin, leptin, and other inflammatory markers (e.g., TNF-*α*) and increases adiponectin levels. The authors explicitly highlight IF as an effective treatment for obesity-related atherosclerosis and a preventive tool (120). Intermittent fasting not only has the potential to benefit the cardiovascular system but is also well tolerated and safe (121). Therefore, when intermittent fasting is combined with standard recommendations, it may also show synergy in reducing the risk of T2DM complications.

**Figure 1:**
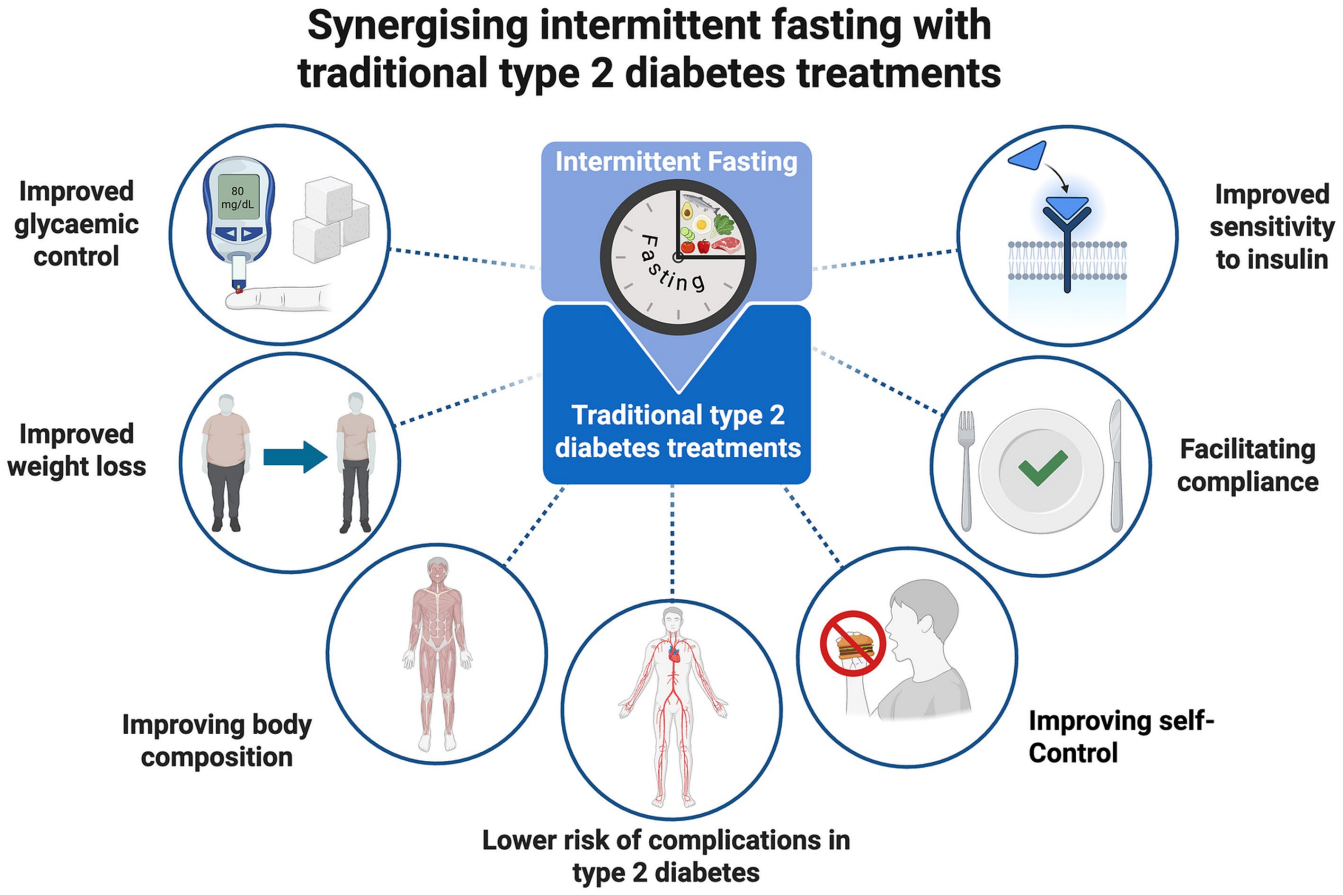
Synergizing intermittent fasting with traditional type 2 diabetes treatments. Created in BioRender. Rodzeń (2025) https://BioRender.com/kik09o6.

Synergizing intermittent fasting with traditional type 2 diabetes treatments is illustrated in Figure 1.

## 5 Efficacy and safety of intermittent fasting in treating type 2 diabetes—study results

Scientific trials focusing on diabetic patients increasingly often look at intermittent fasting in type 2 diabetes. Their number has grown to a point where the first meta-analyses and systematic reviews analyzing the effect of IF in T2DM patients emerge (1, 2, 48, 82, 122). The relevance of scientific evidence is emphatically confirmed by the “Standards of Care in Diabetes-2024,” which considers IF a helpful and safe tool that can be used in both types of diabetes (9). With a vast body of research already available, recommendations can be based on good quality evidence, taking into account randomized controlled trials (RCTs), meta-analyses, and systematic reviews.

### 5.1 Intermittent fasting and type 2 diabetes—randomized controlled trials (RCTs)

A number of randomized controlled trials have investigated the effects of intermittent fasting in patients with T2DM. A study published in 2023 analyzed whether IF was safe and feasible in people with type 2 diabetes treated with insulin. The results demonstrated that IF was not only safe and feasible but also effective. After 12 weeks, patients on IF significantly reduced their glycated haemoglobin levels (by −7.3 ± 12.0 mmol/mol on average) compared to the control group (0.1 ± 6.1 mmol/mol). In addition, the authors examined a composite endpoint consisting of three components: ≥10% reduction in insulin doses, ≥2% reduction in body weight, and ≥3 mmol/mol reduction in HbA1c. The composite endpoint was achieved by 40% of patients in the IF group and none in the control group (7). In the same year, a study on 209 people at increased risk of type 2 diabetes was published. It has found that after 6 months, glucose tolerance (based on an analysis of changes in glucose area under the curve in response to a mixed-meal tolerance test) improved significantly more in patients on intermittent fasting combined with early time-restricted eating (iTRE) than in those on daily calorie restriction (70% of energy requirements) (mean −10.10 vs. −3.57 mg dl −1 min −1), and remained comparable at 18-month follow-up. In view of this, intermittent fasting in the form of iTRE is effective in improving postprandial glucose metabolism in individuals at risk of developing type 2 diabetes (123). In a recent randomized controlled trial (RCT) conducted in 2024, the authors compared the effectiveness of intermittent fasting in the form of time-restricted eating (TRE) with individualized dietary guidance (DIET) provided by a dietitian in patients with type 2 diabetes. The study demonstrated that participants in the TRE group experienced a mean reduction in HbA1c of −0.4%, compared to −0.3% in the DIET group. Body weight decreased by an average of −1.7 kg in the TRE group and −1.2 kg in the DIET group. Notably, self-reported adherence was higher among participants assigned to the TRE intervention (124). In another randomized controlled trial conducted in 2024, researchers compared the change in body weight and metabolic outcomes in patients with type 2 diabetes and obesity undergoing intermittent fasting (IF) in the form of either a 16:8 or 14:10 time-restricted eating regimen, versus a standard control diet. Body weight decreased by −4.02% in the IF 16:8 group, −3.15% in the IF 14:10 group, and −0.55% in the control group. Mean fasting glucose levels declined by −30.91 mg/dL (IF 16:8), −28.06 mg/dL (IF 14:10), and −9.09 mg/dL (control), while mean HbA1c levels decreased by −0.499, −0.528, and −0.197%, respectively. Both IF regimens led to significantly greater reductions in fasting glucose and HbA1c compared to the control group, with no statistically significant difference observed between the two IF schedules (125). In a randomized controlled trial from 2022, patients with type 2 diabetes were divided into two groups: TRE (time-restricted eating) and control. Subjects in the TRE group took their meals within a 10-h daily eating window, while those in the control group had an eating window of at least 14 h. Despite the small difference between the two groups, it appears that the TRE group benefited more compared to the control group in terms of a greater reduction in mean glucose values, both fasting (7.6 ± 0.4 vs. 8.6 ± 0.4 mmoL/L) and during the day (6.8 ± 0.2 vs. 7.6 ± 0.3 mmoL/L), a greater reduction in daily glucose oxidation rate (260.2 ± 7.6 vs. 277.8 ± 10.7 g/day), and even time spent in normoglycemia (15.1 ± 0.8 vs. 12.2 ± 1.1 h per day). The authors conclude that TRE involving eating window restriction to 10 h per day is effective, safe, and feasible in the context of improving glucose homeostasis in patients with type 2 diabetes (126). The authors of another RCT have compared a diet mimicking fasting with energy restriction to 500–600 kcal for 2 days per week (and no restrictions on the other 5 days) against daily energy restriction to 1,200–1,500 kcal. The two diets were tested in patients with type 2 diabetes. Although the first approach is not pure intermittent fasting, it is very close to it. In addition, the trial continued for 12 months, which makes it valuable. Intermittent energy restriction was shown to be as effective as continuous, with similar results in terms of HbA1c, body weight, fasting glucose, and lipid profile. The authors conclude that intermittent energy restriction effectively reduces HbA1c and is comparable to continuous energy restriction in patients with T2DM (127). As a next step, the authors published another article looking at the same patients for 24 months, i.e., 12 months after the intervention was completed. The results of the former participants in both groups were again similar (128). Another RCT indicated that education and reduced medication dosage resulted in fewer (than expected) hypoglycaemic episodes in patients with type 2 diabetes on energy restriction for 2 days per week. In one group, energy was restricted on 2 consecutive days and on 2 non-consecutive days in the other. The authors demonstrated that there was no difference in the incidence of hypoglycaemia between the two groups. Importantly, improvements in fasting glucose, HbA1c, body weight, and even quality of life were recorded in both groups (129). The results of another RCT investigating the effect of intermittent fasting extended for 7 days (with a daily energy allowance of 300 kcal) on people with T2DM showed no adverse effects, and fasting was well tolerated by the patients. The authors conclude that this type of fasting is feasible and may have clinical benefits (130). Yet, another RCT confirms that the effects of 2 days of fasting (with a small amount of calories) per week are similar to those of continuous energy restriction. It is an effective intervention that improves a number of parameters, such as glycaemic control, body weight, and possibly even medication dosage (81).

### 5.2 Intermittent fasting and type 2 diabetes—meta-analyses and systematic reviews

The impact of IF on T2DM has attracted the attention of researchers, resulting in a growing number of published scientific studies. This, in turn, has brought about more comprehensive publications in the form of meta-analyses and systematic reviews.

In 2025, a systematic review was published evaluating the effects of intermittent fasting (IF) and continuous caloric restriction (CCR) on glycemic control and weight reduction in individuals with type 2 diabetes, based on evidence from randomized controlled trials and observational studies conducted primarily between 2000 and 2024. While both dietary strategies demonstrated certain limitations, the review highlighted several notable short-term benefits of IF, including significant reductions in HbA1c levels, fasting glucose concentrations, and body weight—outcomes that are particularly relevant in the management of type 2 diabetes (131). In support of these findings, a meta-analysis published in 2025 confirmed the efficacy and superiority of intermittent fasting (IF) over control conditions not only in reducing fasting glucose levels, HbA1c, and body weight, but also in improving body mass index (BMI), waist circumference, systolic and diastolic blood pressure, low-density lipoprotein (LDL) cholesterol, and total cholesterol. No significant differences were observed in postprandial glucose, high-density lipoprotein (HDL) cholesterol, or triglyceride levels. The authors concluded that IF is an effective strategy for lowering glucose levels in patients with type 2 diabetes, while also being both feasible and safe for clinical implementation (132). Another meta-analysis, including a total of 1,101 adult participants with type 2 diabetes or prediabetes, demonstrated that intermittent fasting (IF) led to significant reductions in body weight (−4.56 kg), body mass index (−1.99 kg/m^2^), HbA1c (−0.81%), fasting glucose (−0.36 mmol/L), total cholesterol (−0.31 mmol/L), and triglycerides (−0.14 mmol/L), without exerting a significant impact on fat mass, insulin levels, LDL, HDL, or blood pressure compared to control groups (133). The authors of a 2023 systematic review demonstrated (based on limited evidence) that intermittent energy restriction (IER) and periodic fasting (PF) in patients with T2DM may be beneficial for metabolic health parameters and anthropometric parameters. It may even lead to reduced dosage of hypoglycaemic drugs (2). Another publication evaluated the effect of IF on metabolic homeostasis control. Although the publication focused on a broader range of metabolic diseases, it was observed that, specifically in patients with type 2 diabetes, IF was beneficial in the context of insulin homeostasis, among other things. The same publication demonstrated a range of benefits of different types of fasting on metabolic health (39). Another publication, also covering a broader range of metabolic risk factors, compared IF with daily caloric restriction (DCR). Based on limited evidence, it showed that IF may even be more effective than DCR for improving insulin sensitivity and fat loss. In terms of other chronic disease risk factors and weight loss, IF was as effective as DCR (41). A number of subsequent meta-analyses and systematic reviews confirm the efficacy of intermittent fasting, highlighting its beneficial effects on metabolic health (also in aspects closely related to type 2 diabetes) (134–138). Interestingly, since fasting can improve glucometabolic markers even in healthy individuals (139), it is reasonable to believe that it is helpful in preventing type 2 diabetes. The authors of a 2022 meta-analysis conclude that both intermittent fasting and the ketogenic diet (KD) can help treat type 2 diabetes through glycaemic control and weight loss (140). Another meta-analysis showed no statistically significant difference between IF-mediated glucose level reduction and reduction attributable to other forms of intervention (e.g., continuous energy restriction). However, the study suggests that IF may be beneficial in long-term glycaemic control or even insulin sensitization. The authors conclude that intermittent fasting may be an effective preventive tool against type 2 diabetes in a pre-diabetic population (1). Interesting results are presented in a 2023 systematic review with a meta-analysis, which assessed the effect of intermittent fasting in the form of Ramadan on glycaemic control in patients with type 2 diabetes. The focus was on two key parameters, i.e., glycated haemoglobin and fasting glucose, both assessed before and after Ramadan. It was found that both HbA1c and fasting glucose decreased significantly after fasting during Ramadan. HbA1c (%) was reduced significantly, by an average of 0.55, and fasting glucose by an average of 12.42 mg/dL (48). The authors of another article set out to compare the safety of intermittent fasting against continuous energy-restricted diets (CERDs) in people with type 2 diabetes and metabolic syndrome. They found that both IF and CERD had beneficial effects on fasting glucose and insulin, HbA1c, and lipid profile. Furthermore, IF was demonstrated to be superior even with regard to weight loss. In view of the evidence reviewed, it was concluded that intermittent fasting was safe and could be used in patients with type 2 diabetes or metabolic syndrome (82). Another review covered 68 studies (including 35 RCTs) and focused on the effect of intermittent fasting during Ramadan in the context of risks associated with hypoglycaemic episodes (HEs) in T2DM patients. The authors point out that to prevent HE, adjusting medication dose is often necessary, along with monitoring the patient’s condition. Patient education and awareness are essential as well (141). It is therefore important to realize what a powerful tool fasting can be, given that many patients with type 2 diabetes need to reduce their medication dosage. Another meta-analysis demonstrated that intermittent fasting was superior to a standard diet (control groups) in people with T2DM. IF worked better in terms of weight loss (and excessive weight often accompanies type 2 diabetes) and was equally effective for glycaemic control. The therapeutic potential of IF attributable to weight loss in patients with type 2 diabetes is highlighted (122). Finally, a 2020 systematic review focused on the effect of IF on glycaemic control and body composition in patients with T2DM and obesity. Intermittent fasting was shown to be feasible and effective in improving glycaemia and body composition within 12–24 weeks (142).

The evidence discussed above demonstrates the benefits of intermittent fasting in type 2 diabetes, which sometimes are in fact superior (and rarely inferior) to those of continuous energy restriction.

## 6 Novel technologies for safe and effective intermittent fasting

Novel technologies can make intermittent fasting simpler, safer, and more effective for people with type 2 diabetes. They primarily include glucose monitoring devices and mobile apps that help patients adhere to intermittent fasting.

### 6.1 Glucose monitoring devices

Glucose monitoring during intermittent fasting seems particularly important for people with type 2 diabetes. Indeed, in individuals with disturbed carbohydrate metabolism who frequently take hypoglycaemic drugs, prolonged abstinence from food can lead to episodes of hypoglycaemia (129, 141). Continuous glycaemic monitoring seems very helpful in keeping track of the body’s response to IF and thus avoiding hypoglycaemia. Continuous Glucose Monitoring (CGM) technologies allow glucose levels to be monitored in real time, day and night. CGM devices consist of a glucose sensor inserted in the skin (secured with an adhesive patch) and a transmitter that sends blood glucose information to a device, which can be an ordinary smartphone (143). By enabling better glycaemic control, the technology is particularly recommended for people suffering from hypoglycaemic episodes, hyperglycaemia, and other glucose disorders. The benefits of CGM are well supported by published evidence. Therefore, the technology is recommended by national and international medical organizations and expert consensus in type 2 diabetes, type 1 diabetes, and women with gestational diabetes. CGM literacy is even suggested to be important in the context of medical examination of patients applying for disability allowance due to diabetes (144–146). CGM appears to be helpful for people with type 2 diabetes during intermittent fasting.

### 6.2 Mobile apps

A large number of mobile apps monitor health status and help users make better dietary and lifestyle choices. In addition, rapid progress in the development of artificial intelligence means that new mobile app functionalities can assist patients even further. Scientific findings from studies linking mobile apps and intermittent fasting are already available. A study by Valinskas et al. (147) investigated the effectiveness of IF recommendations delivered via a mobile app in the context of weight loss and user engagement. The authors found that active app users lost significantly more weight (which also translated into BMI) compared to inactive users. The number of days of activity and total duration of app usage were shown to be the most important weight loss predictors (147). Another study also concluded that app-assisted intermittent fasting was a promising strategy for weight loss in overweight and obese individuals. Interestingly, greater weight loss is correlated with greater fasting frequency, alongside initial BMI and the number of hours of fasting per day. The authors point out certain factors, such as diet, physical activity, stress, and smoking, that can be successfully tracked using mobile apps (148), thus allowing the user to monitor their progress and helping them reach their goals.

It is also known that such apps can be effective in patient education on any given topic, including intermittent fasting. The promotion of health education itself using different platforms, including smartphone apps, has also been suggested in the literature (149), even among medical professionals (150). Apps can provide users with information on healthy lifestyles, proper nutrition, and the benefits of regular intermittent fasting.

**Figure 2:**
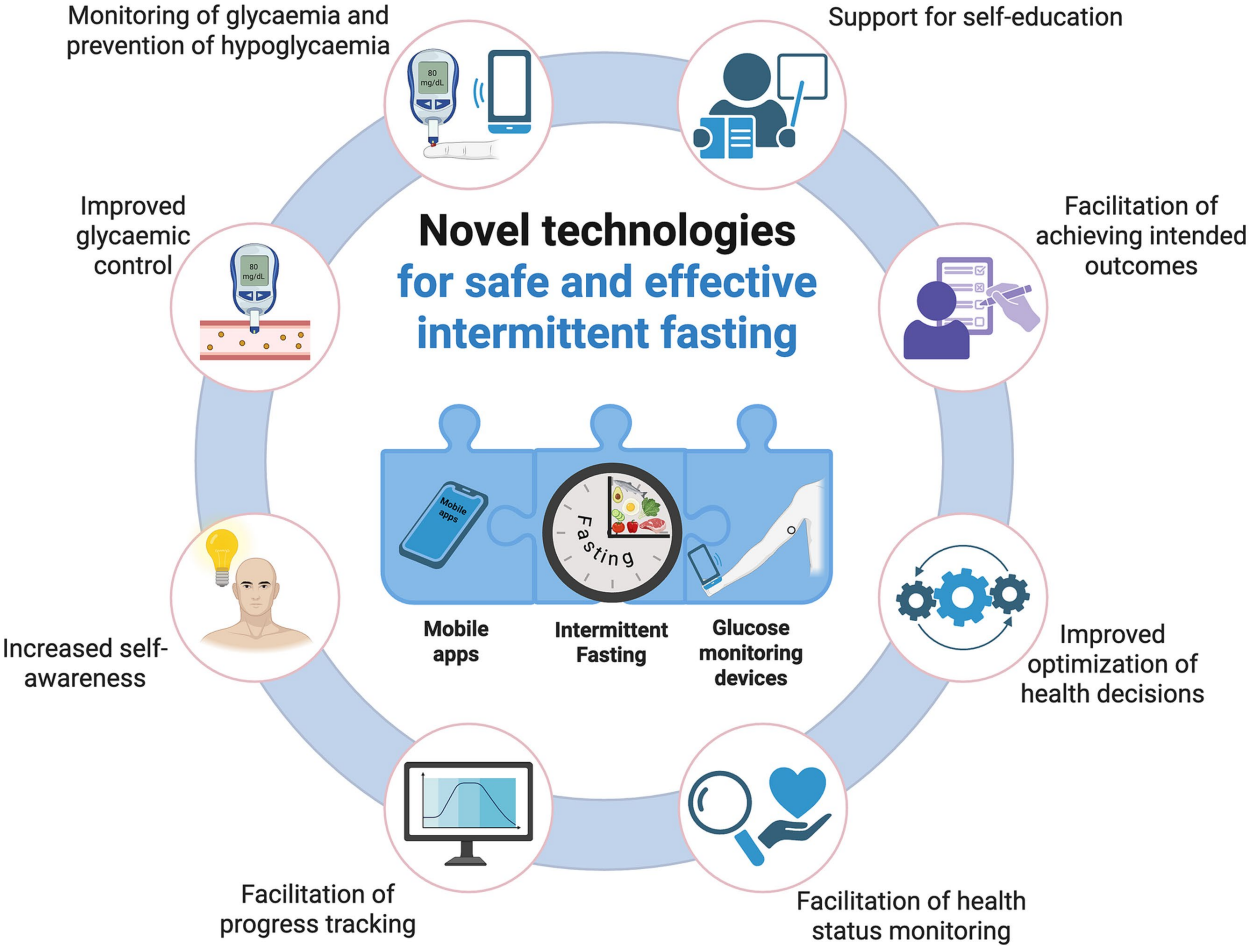
Novel technologies for safe and effective intermittent fasting. Created in BioRender. Rodzeń (2025) https://BioRender.com/7hws22e.

In addition, the rapidly advancing predictive modeling, if introduced into mobile apps, could predict the body’s response to a given agent based on the patient’s historical data and current health status (and other factors) (151, 152). Similarly, it would be possible to develop an app that predicts (based on the history of a patient with type 2 diabetes) the body’s potential response to intermittent fasting. In this way, early prevention of hypoglycaemic episodes would be possible. Novel technologies for safe and effective intermittent fasting are illustrated in Figure 2.

## 7 Intermittent fasting is not for everyone

Intermittent fasting (IF) may offer promising health benefits in various populations; however, it is not universally appropriate and should be considered with caution in certain clinical contexts. For example, IF is generally not recommended for individuals with eating disorders, who often exhibit significant disruptions in eating behaviors and require individualized, structured therapeutic approaches. In such cases, implementing IF may exacerbate underlying psychopathology or perpetuate maladaptive food-related patterns (153, 154). Similarly, in pregnant women, certain forms or patterns of IF have been associated with adverse maternal and offspring health outcomes, including fatigue, dehydration, and potential impacts on fetal development (155, 156). While some studies suggest potential benefits of controlled IF during pregnancy (156), the risk–benefit ratio should be carefully evaluated, and if IF is implemented, it should be under close professional supervision. The postoperative recovery period also appears suboptimal for initiating IF protocols. During this time, patients often require tailored nutritional support to facilitate healing and maintain hydration and energy balance; the physiological stress of recovery may render fasting counterproductive (157). Caution is also warranted when considering IF in elderly individuals, particularly those at risk of unintended weight loss or malnutrition. Similarly, patients who are underweight, those prone to compensatory overeating (e.g., food reward after fasting), or individuals on certain pharmacologic agents may face increased risks when engaging in fasting regimens (158). Moreover, IF is contraindicated in patients with specific rare metabolic or genetic disorders, such as pyruvate carboxylase deficiency (PCD) (159), primary carnitine deficiency (PCD) (160), carnitine-acylcarnitine translocase (CACT) deficiency (161), 3-hydroxyacyl-CoA dehydrogenase deficiencies, including both medium-chain (MCHAD) and long-chain (LCHAD) forms (162, 163), medium-chain acyl-CoA dehydrogenase deficiency (MCADD) (164), very-long-chain acyl-CoA dehydrogenase deficiency (VLCADD) (165), and porphyria (166). In light of these considerations, a personalized risk assessment is essential before initiating IF, especially in vulnerable populations or those with complex medical conditions.

## 8 Limitations

The studies discussed have certain limitations. One of these is the variability in study duration, with some studies being short-term and others long-term. Another limitation is the diversity of populations, as the studies do not focus on a single, specific population but rather exhibit a broad range of demographic characteristics. Additionally, although most studies demonstrate a certain degree of consistency, it cannot be claimed that all of them are entirely consistent. Some studies do not show significant benefits of intermittent fasting (IF) for patients with type 2 diabetes. However, it should be clearly noted that this is most likely due to the diversity of methodologies used across the studies, as well as the type and duration of intermittent fasting, the total daily caloric intake, and the specific populations in which it was applied.

Furthermore, this study does not focus on the use of intermittent fasting (IF) in younger populations. There is limited evidence regarding the application of IF in children and adolescents, primarily due to the relatively low, albeit increasing, prevalence of type 2 diabetes mellitus (T2DM) in this group. The feasibility, acceptability, and safety of IF in children and adolescents require further investigation, as their nutritional, growth, and safety needs differ from those of adults.

## 9 Final remarks

Based on the above, it is certainly reasonable to conclude that intermittent fasting can be an effective and safe tool in the treatment of type 2 diabetes. It appears that IF is an interesting (and synergistic) addition to the standard T2DM recommendations, thus improving the patients’ health. This is owed to its intrinsic properties, particularly desirable in conditions affecting patients with T2DM. First and foremost, IF is effective in improving glycaemic control. It is obvious that a period of fasting, with no energy supply from food and drink, is equivalent to the absence of glucose and insulin spikes during this time. If intermittent fasting continues for 16 h per day, then the potential exposure to glucose and insulin spikes is only during the remaining 8 h (the daily eating window), and is therefore limited to only ⅓ of the day. The effectiveness of IF in stabilizing glycaemia has been confirmed by the studies discussed in Section 4.1. Another advantage is the improvement of the body’s insulin sensitivity, thus reducing insulin resistance, the key underlying cause of type 2 diabetes. In this regard, intermittent fasting is helpful in a number of ways described in detail in Chapter 4.2. IF is also undoubtedly useful in facilitating compliance. Eating meals over an 8-h window is often less problematic than eating five to six meals from morning to evening. This applies to the time spent on cooking and consumption on the one hand, and the challenge of keeping an eye on the appropriate intervals between the meals on the other. In addition, if the eating window is longer, meals must often be eaten in an unfavorable setting (e.g., at work). Thus, IF can be very helpful, allowing patients to focus on the quality rather than the quantity of their meals. Another aspect associated with IF is weight loss, which in some ways is also linked to the other beneficial properties of fasting in diabetes. This can be described as a cause-and-effect sequence that exists in addition to the effect of the individual factors separately. Namely, intermittent fasting promotes lower energy intake (in a shorter daily eating window, the risk of overeating is lower); this results in weight loss, which in its turn improves glycaemic and insulin parameters (which are inter-related); together with the weight loss itself, this helps manage inflammation (reduction in pro-inflammatory cytokines), improves the lipid profile and the endothelial function, and indirectly reduces the risk of complications (including the particularly common cardiovascular incidents) in patients with type 2 diabetes. All of these factors can interact and should be analyzed in the context of their synergistic effect.

Importantly, the results of studies looking at the impact of IF on patients with type 2 diabetes are clearly promising. Nevertheless, potential risks, mainly caused by possible hypoglycaemic episodes, should be borne in mind as well. Intermittent fasting is such a powerful tool in terms of lowering glycaemia that, in some patients, the dose of hypoglycaemic drugs needs to be adjusted (often significantly reduced). This issue must not be overlooked, as the authors of the studies cited in this review point out. It is important to emphasize that not all patients with type 2 diabetes mellitus are obese, and the response to intermittent fasting may differ between lean and obese individuals. This distinction could play a significant role when designing future studies or clinical recommendations. Age-related differences in the outcomes and risks associated with IF should also be considered. The effectiveness and safety of IF may vary between younger and older populations, with further research needed to address these age-specific differences.

Although intermittent fasting dates back to antiquity, novel technologies can further improve its use. Continuous glucose monitoring (CGM) devices can help keep track of the body’s response to fasting, which is particularly important with regard to potential hypoglycaemia, as it enables early intervention. In turn, mobile apps are useful for preparing a fasting routine, counting fasting hours, recording patient data, or providing patient education on intermittent fasting.

## 10 Conclusion

If properly adhered to, intermittent fasting can be an effective and safe tool in the treatment of type 2 diabetes. It can work synergistically with official recommendations for T2DM patients, thus increasing their effectiveness and improving the management of the disease. Mobile apps and CGM (Continuous Glucose Monitoring) devices may be helpful in practicing IF. Vigilance on possible hypoglycaemic episodes, especially in patients taking hypoglycaemic drugs, is important. More emphasis on further research in this area is clearly necessary, as all indications are that IF in T2DM is a promising direction.
